# Co-Occurrence of Myeloid and Lymphoid Neoplasms: Clinical Characterization and Impact on Outcome. A Single-Center Cohort Study

**DOI:** 10.3389/fonc.2021.701604

**Published:** 2021-10-18

**Authors:** Cristina Bucelli, Bruno Fattizzo, Daniele Cattaneo, Juri Alessandro Giannotta, Kordelia Barbullushi, Raffaella Pasquale, Enrico Barozzi, Maria Chiara Barbanti, Loredana Pettine, Francesca Gaia Rossi, Gianluigi Reda, Ramona Cassin, Wilma Barcellini, Luca Baldini, Alessandra Iurlo

**Affiliations:** ^1^ Hematology Unit, Fondazione IRCCS Ca’ Granda Ospedale Maggiore Policlinico, Milan, Italy; ^2^ Department of Oncology and Onco-Hematology, University of Milan, Milan, Italy

**Keywords:** myeloproliferative neoplasms, lymphoproliferative syndromes, myelodysplastic syndromes, infections, secondary malignancies

## Abstract

The co-occurrence of myeloid neoplasms and lymphoproliferative diseases (LPDs) has been epidemiologically described, particularly in myeloproliferative neoplasms (MPNs). However, the clinical features of these patients are poorly known. In this study, we evaluated a single-center cohort of 44 patients with a diagnosis of myeloid and LPD focusing on clinical features, therapy requirement, and outcome. The two diagnoses were concomitant in 32% of patients, while myeloid disease preceded LPD in 52% of cases (after a median of 37 months, 6–318), and LPD preceded myeloid neoplasm in 16% (after a median of 41 months, 5–242). The most prevalent LPD was non-Hodgkin lymphoma (50%), particularly lymphoplasmacytic lymphoma (54.5%), followed by chronic lymphocytic leukemia (27%), plasma cell dyscrasias (18.2%), and rarer associations such as Hodgkin lymphoma and Erdheim–Chester disease. Overall, 80% of *BCR-ABL1*-negative MPN patients required a myeloid-specific treatment and LPD received therapy in 45.5% of cases. Seven subjects experienced vascular events, 13 a grade >/= 3 infectious episode (9 pneumonias, 3 urinary tract infection, and 1 sepsis), and 9 developed a solid tumor. Finally, nine patients died due to solid tumor (four), leukemic progression (two), infectious complications (two), and brain bleeding (one). Longer survival was observed in younger patients (*p* = 0.001), with better performance status (*p* = 0.02) and in the presence of driver mutations (*p* = 0.003). Contrarily, a worse survival was significantly associated with the occurrence of infections (*p* < 0.0001). These data suggest that in subjects with co-occurrence of myeloid and lymphoid neoplasms, high medical surveillance for infectious complications is needed, along with patient education, since they may negatively impact outcome.

## Introduction

The potential for myeloid neoplasms to evolve one into each other is largely known [i.e., leukemic evolution of myeloproliferative neoplasms (MPNs) and myelodysplastic syndromes (MDSs)], and the same occurs for lymphoproliferative disorders [(LPDs), i.e., chronic lymphocytic leukemia, (CLL), which may evolve to aggressive non-Hodgkin lymphomas (NHLs)]. Less is known about the permutation of a myeloid into a lymphoid neoplasm and vice versa, and about their co-occurrence. Some reports describe epidemiological associations of myeloid and lymphoid cancers, and a large Italian study involving 820 MPN patients reported an increased risk for LPD (3.4-fold greater for CLL and 12.4-fold for NHL) and solid tumors compared to the general population (1). From a lymphoid perspective, CLL patients are known to be at higher risk for secondary neoplasms, mostly cutaneous ones (2); however, hematological neoplasms are rarely observed. Irrespective of the former neoplasm (either myeloid or lymphoid), important concerns have been raised about the possible causal effect of hematological treatments on the development of second tumors, and it is still an unanswered question. Moreover, little is known about the clinical characteristics of patients with co-occurrence of myeloid and lymphoid neoplasms, and their outcome in terms of infectious and thrombotic complications, and survival. In this study, we evaluated a single-center cohort of patients with a double diagnosis to assess their clinical features, therapy requirement, and outcome.

## Patients and Methods

We retrospectively evaluated a cohort of 1,351 myeloid neoplasms (930 MPNs and 421 MDSs) diagnosed within the period 1987–2020 and followed up at a tertiary hematological center in Milan, Italy.

All patients displaying the association of a myeloid and lymphoid neoplasm were included (either presenting with myeloid, lymphoid, or concomitant diseases).

MPN diagnoses, including polycythemia vera (PV), essential thrombocythemia (ET), primary myelofibrosis (PMF), chronic myeloid leukemia (CML), and myeloproliferative neoplasms, unclassified (MPN, U), were made according to the most recent WHO classification and current guidelines (3). The same was performed for MDS cases.

Lymphoid diseases included NHL, Hodgkin lymphomas (HLs), CLL, and plasma cell dyscrasias (PCDs), with a histological diagnosis according to current guidelines (4).

For each patient, we evaluated clinical features at first diagnosis, including demographics, performance status according to the Eastern Cooperative Oncology Group scale (ECOG), hematological parameters, cytogenetic abnormalities, and the presence of driver mutations (*JAK2*V617F, *CALR*, and *MPL*) for MPN cases.

The time from initial diagnosis to LPD development and LPD type was collected and all therapies performed for both myeloid and lymphoid disorders. For LPDs, the time from diagnosis to first treatment was also calculated.

Concerning outcome, the occurrence of thrombosis, infections, and death was registered and overall survival (OS) analyzed.

For statistical analysis, Student’s t-test or Wilcoxon test was used for continuous variables where appropriate. Chi-squared or Fisher’s exact tests were used for the comparison of categorical variables, where appropriate. Analysis of variance was performed by using mean, median, ranges, and standard errors. Once identified, variables associated with occurrence of complications and OS hazard ratios for 95% confidence intervals were calculated by Cox regression models. Overall survival was evaluated by Kaplan–Meier method.

## Results

### Baseline Features

During a median follow-up of 9 years (range, 0.8–35), a total of 44 of the 1,351 patients (3.25%) were diagnosed with both a myeloid and lymphoid neoplasm. The two diagnoses were concomitant in 32% of patients, while myeloid disease preceded LPD in 52% of cases after a median time of 37 months (range, 6–318) from myeloid disease, and LPD preceded myeloid neoplasm in 16% after a median time of 41 months (range, 5–242). **Table 1** shows clinical and laboratory characteristics: patients were mainly male, elderly (61% aged >65 years), with a good performance status (96% ECOG 0–1), and all but three had a diagnosis of MPN (3 out of 158 CMLs and 38 out of 772 *BCR-ABL1*-negative MPNs). The remaining subjects were low-risk MDS with multilineage dysplasia (3/421 total MDS patients). Cytogenetic aberrations, excluding t(9;22), were reported in nine patients, comprising one complex karyotype. Among *BCR-ABL1*-negative MPN patients, *JAK2*V617F mutation was found in 30 (79%) cases, while 8% and 3% were *CALR* and *MPL* mutated, respectively. In those first presenting with a myeloid disease, mutated patients showed a longer time to LPD development (mean 120 ± 92 vs. 31 ± 17 months in triple-negative cases; *p* = 0.01). As shown in **Table 2**, the most prevalent LPD was NHL (50%), all but one of B-cell origin, particularly lymphoplasmacytic lymphoma (54.5%). Most NHL were indolent, except for one diffuse large B-cell lymphoma (DLBCL). The second most frequent LPD was CLL (27%), followed by plasma cell dyscrasias (18.2%), and rarer associations such as HL and Erdheim-Chester disease.

**Table 1:** Clinical and hematological characteristics of patients with concomitant myeloid and lymphoid neoplasms.

Table 1	All patients (N = 44)
**Median age, years (range)**	70 (21–93)
**Males**	25 (56)
**PS ECOG**	
**0**	32 (73)
**1**	10 (23)
**2**	1 (2)
**3**	1 (2)
**Laboratory data at diagnosis, median(range)**	
**Leukocytes, ×10^9^/L**	9.56 (1.8–116)
**Hemoglobin, g/dl**	14 (7–20)
**Platelets, ×10^9^/L**	489 (20–1482)
**Lymphocytes, ×10^9^/L**	1.94 (0.69–25)
**Sequence of neoplasm**	
**First myeloid**	23 (52)
**Concomitant**	14 (32)
**First lymphoid**	7 (16)
**Myeloid type**	
**ET**	8 (18)
**PV**	12 (27)
**PMF**	13 (30)
**MPN, U**	5 (11)
**CML**	3 (7)
**MDS**	3 (7)
**Splenomegaly**	22 (50)
**Driver mutations**	
***JAK2*V617F**	30 (79)
**Median allele burden, % (range)**	28.7 (1.2-97.3)
***CALR***	3 (8)
***MPL***	1 (3)
**Triple-negative**	4 (10)
Cytogenetic aberrations*	9 (21)
**LPD type**	
**NHL**	24 (55)
**CLL**	11 (25)
**HL**	1 (2)
**PCD**	8 (18)
**Complications**	
**Thrombosis**	7 (16)
**Infections**	13 (30)
**Solid tumors**	9 (21)
**Death**	9 (21)

Values are given as N (%) unless otherwise specified.

*Excluding CML.

PS ECOG, performance status according to the Eastern Cooperative Oncology Group; ET, essential thrombocytemia; PV, polycytemia vera; PMF, primary myelofibrosis; MPN U, myeloproliferative neoplasm unclassified; CML, chronic myeloid leukemia; MDS, myelodysplastic syndrome; LPD, lymphoproliferative disorder; NHL, non-Hodgkin lymphoma; CLL, chronic lymphocytic leukemia; HL, Hodgkin lymphoma; PCD, plasma cell dyscrasia.

**Table 2:** Overview of the associated myeloid and lymphoid malignancies.

	PV	ET	PMF	CML	MPN-U	MDS	Total
**Chronic lymphocytic leukemia**	2	3	5	1	1	–	12
**Lymphoplasmocytic lymphoma**	4	2	2	–	3	–	11
**Follicular lymphoma**	1	1	3	–	–	–	5
**Marginal zone lymphoma**	–	–	2	–	–	2	4
**Diffuse large B-cell lymphoma**	1	–	–	–	–	–	1
**Multiple myeloma**	3	1	1	1	–	1	7
**Plasmocytoma**	–	1	–	–	–	–	1
**Erdheim**–**Chester disease**	1	–	–	–	–	–	1
**Mycosis fungoides**	–	–	–	–	1	–	1
**Hodgkin lymphoma**	–	–	–	1	–	–	1
**Total**	12	8	13	3	5	3	44

ET, essential thrombocytemia; PV, polycytemia vera; PMF, primary myelofibrosis; MPN U, myeloproliferative neoplasm unclassified; CML, chronic myeloid leukemia; MDS, myelodysplastic syndrome.

### Therapy Requirement

The clinical characteristics and therapy sequences of patients divided according to the first presenting neoplasm are detailed in the **Supplementary Table**. Overall, 80% of *BCR-ABL1*-negative MPN patients required myeloid-specific treatment, including hydroxyurea in most cases (71%), followed by ruxolitinib in three patients, and pipobroman in two. Of note, 14 cases had been treated before LPD diagnosis (11 hydroxyurea, 1 hydroxyurea and pipobroman, and 2 ruxolitinib), 11 cases started therapy concomitantly (10 hydroxyurea, 1 ruxolitinib, and 1 imatinib), and 11 after LPD diagnosis (9 hydroxyurea, 1 hydroxyurea and imatinib, and 1 hydroxyurea and pipobroman). All CML patients received imatinib either before, concomitantly, or after LPD diagnosis (one case each). Moreover, 29 patients were on antiplatelet prophylaxis (27 aspirin and 2 ticlopidine). Finally, one MDS subject received recombinant erythropoietin and steroids (concomitant MDS and LPD diagnosis). Lymphoid diseases required specific treatment in 45.5% of cases, after a median time from the first diagnosis of 8 months (range, 0–115). The following therapies were administered: seven anti-CD20 monoclonal antibody plus chemotherapy, one rituximab-ibrutinib, three chemotherapies, two radiotherapies, one pegylated interferon, one parotidectomy, one phototherapy, two steroids only, one lenalidomide-dexamethasone, and one allogeneic hematopoietic stem cell transplant. Notably, four patients had received LPD treatment before myeloid diagnosis.

### Complications

Seven patients (4 ETs and 3 PMFs) experienced a total of 10 vascular events (5/7 occurring before diagnosis of the second hematological neoplasm), including 7 venous thromboses (3 pulmonary embolisms, 1 cerebral vein thrombosis, 1 retinal vein thrombosis, and 2 portal vein thromboses), and 3 arterial events (1 myocardial infarction, 1 stroke, and 1 ileal infarction). Of note, three patients experienced more than one events. All venous events were managed with low molecular weight heparin, and four were switched to long-term oral anticoagulants (three warfarin and one on direct oral anticoagulant). Unexpectedly, *JAK2* resulted unmutated in more than half of the cases (57.1%), and no associations were observed with LPD type.

Concerning infections, 13 (30%) patients experienced a grade ≥3 episode, including 9 pneumonias (of whom 2 fatal), 3 urinary tract infection, and 1 sepsis, all due to bacterial agents (12/13 occurring after the second neoplasm diagnosis). Infections were mainly diagnosed in elderly subjects (12/13, *p* = 0.006), in CLL patients (54%), and only one after a recent LPD therapy with rituximab plus chemotherapy. Importantly, no patients were on ruxolitinib at the time of infection.

After a median time from hematological diagnosis of 106 months (range, 0–301), nine subjects (five PMFs, two CMLs, one PV, and one ET) developed a solid tumor (two lung, two gastric, one liver, one kidney, one cutaneous, one bladder carcinoma, and one seminoma); six occurred after the second hematological diagnosis (**Supplementary Table**). Of note, one patient had a concomitant diagnosis of PMF, follicular lymphoma, CLL, and lung cancer. Overall, cases developing a solid tumor had a shorter time to first LPD therapy as compared to those without solid cancer (7 ± 10 vs. 37 ± 42 months; *p* = 0.02).

### Survival

**Figure 1:**
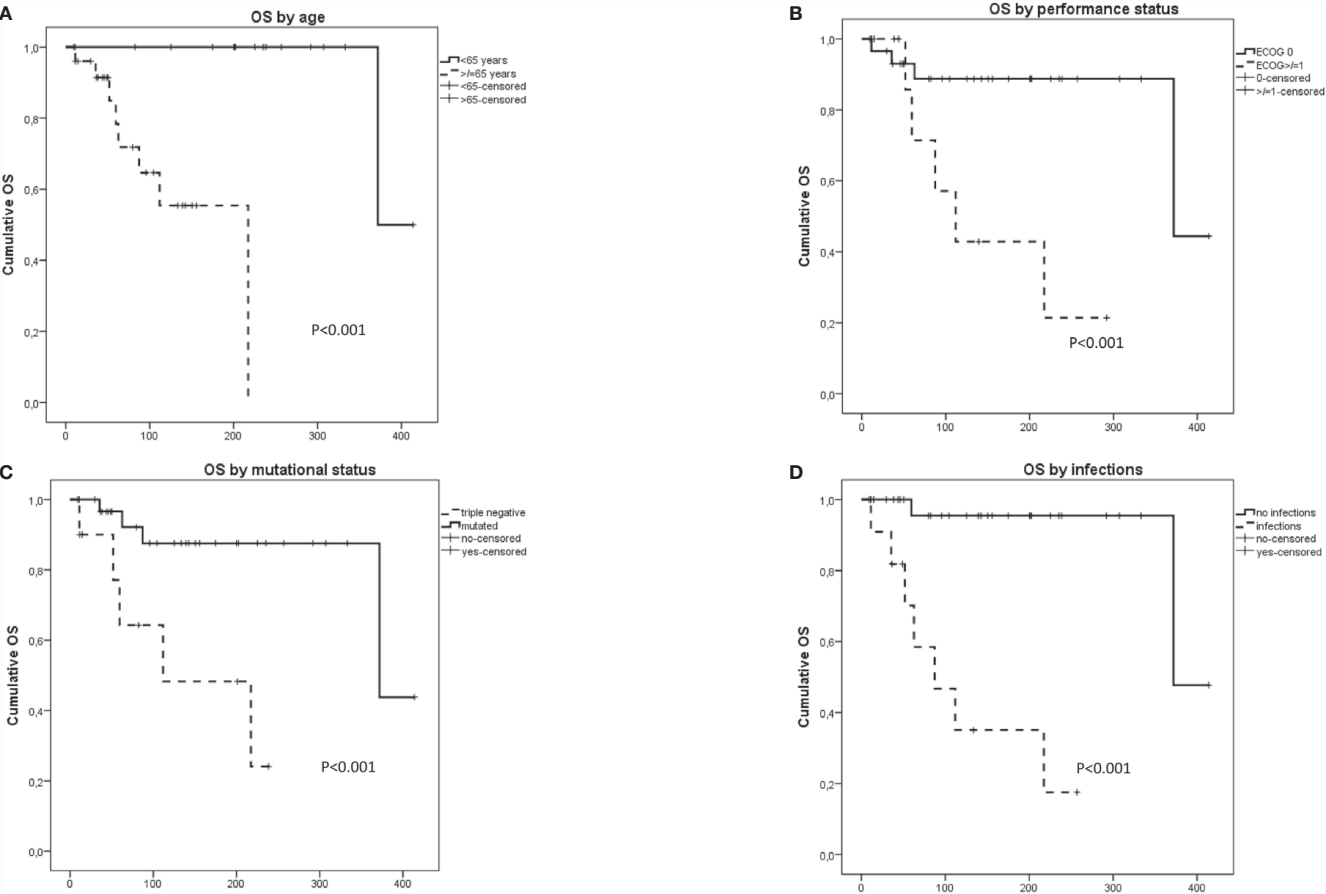
Overall survival in patients with concomitant myeloid and lymphoid neoplasms according to **(A)** age, **(B)** performance status, **(C)** presence of driver mutation, and **(D)** infectious complications.

Nine (20%) patients died due to solid tumor (four), leukemic progression (two), infectious complications (two), and brain bleeding (one). Fatalities were more frequent in patients with lymphoid treatment requirement (35% vs. 9%; *p* = 0.04). As shown in **Figure 1**, a longer OS was observed in younger patients (*p* = 0.001), with better performance status (*p* = 0.02) and in the presence of driver mutations (*p* = 0.003). Contrarily, a worse survival was significantly associated with the occurrence of infections (*p* < 0.0001). Multivariate analysis by Cox regression model showed that the occurrence of infections was the only independent predictor of worse survival within the cohort (HR, 3.18; 95%CI, 2.9–19; *p* = 0.003).

## Discussion

Here, we describe a single-center series of associated myeloid and lymphoid neoplasms and contribute to delineate some peculiar clinical features and their impact on outcome. In particular, these patients, despite being mainly chronic/indolent neoplasms (i.e., MPN, low-risk MDS, and indolent LPD), display a dismal outcome, mostly related to infectious complications. Importantly, the detrimental impact of infections on survival seems independent from LPD aggressiveness or therapy. In fact, only one infection was related to recent rituximab plus chemotherapy and none to ruxolitinib. This might hint that the infectious risk is more disease intrinsic than iatrogenic, is possibly favored by the presence of a double myeloid/lymphoid clonality, and should be considered even in untreated patients. Interestingly, infections had a higher impact on survival than thrombotic episodes that are the major cause of morbidity and mortality in MPNs. In this study, thromboses were not associated with *JAK2* mutation in MPN-LPD cases, contrarily to what was reported for classic MPNs (5, 6). Moreover, most events occurred in patients presenting with myeloid neoplasm, before the development of the LPD, and did not have an impact on survival. Additionally, together with expected dismal outcome in elderly patients with poorer performance status, triple negative MPN-LPD subjects also showed shorter OS, as already described for isolated PMF (7). Triple negative MPN-LPD cases also displayed a shorter time to LPD development compared to mutated ones, although this observation requires further investigation.

From an epidemiological point of view, an Italian population study has reported that MPN patients (excluding CML) have a 3.44-fold increased risk of LPD compared with the general population (1). This was confirmed by a further review of 1,915 MPN patients of whom 22 displayed coexistent LPD, with a calculated risk 2.79-fold higher than the general Italian population (8). Our results well compare to these data, with a prevalence of 3.25%, although a direct comparison with the general population has not been performed. At variance, other studies reported a higher prevalence of myeloid/lymphoid associations (up to 15%), possibly due to the inclusion of pre-neoplastic conditions such as monoclonal gammopathy of uncertain significance (MGUS) and monoclonal B-cell lymphocytosis (MBL) (9–11).

In our series, a minority of patients had LPD preceding myeloid neoplasm, mostly CLL or indolent NHL, while plasma cell dyscrasias and aggressive neoplasms occurred all concomitantly or after myeloid diagnosis. This finding is in accordance with a recent meta-analysis, where aggressive forms were rarer (14%), mostly concomitant or subsequent to myeloid cancer diagnosis, and required chemotherapy in 7% of cases only (12).

Biologically, it has been speculated that the co-occurrence of myeloid and lymphoid neoplasms may be sustained by a common clonal progenitor (13, 14). This could be assumed particularly in MPN patients with a demonstrated driver mutation, preceding LPD onset. In our series of MPN-LPD cases, *JAK2*V617F was overrepresented as compared to MPN patients not developing lymphoid diseases (79% vs. 65%). Consistently, it has been shown that patients harboring the *JAK2*V617F mutation display an increased risk (5.46-fold) of LPD development (1, 15). Moreover, Vannucchi *et al.* demonstrated the *JAK2*V617F mutation in lymphoid tumor cells in two out of three evaluated cases (1). Conversely, the presence of a “true” double clonality might be hypothesized, with the myeloid and lymphoid clones facilitating each other’s selection/expansion in a vicious circle. This could be in line with our study and that of others, reporting the possible coexistence or subsequent development of the two diseases and with the hardly demonstrable common origin of the two clones in most cases. Finally, the possible contribution of germline mutations, including DDX41, RUNX1, ETV6, ANKRD26, and POT1, which have been associated with the occurrence of both myeloid and lymphoid neoplasms, is an intriguing point to be explored in future studies (3, 16).

External triggers, such as hematological therapies, may also favor the emergence of the second clone. In our series, the only therapies administered before LPD development were hydroxyurea, ruxolitinib, and pipobroman. It has been speculated that these drugs may contribute to a reduced immune surveillance on the development of second tumors (17, 18). This immunological derangement is already present in untreated MPN patients and encompass the increase in myeloid-derived suppressor cells and the defective function of T-regulatory and NK cells, showing impaired degranulation and killing capacity. Ruxolitinib may further exert a negative influence on the innate and the adaptive immune system (19). Accordingly, it has been reported that its use may disclose a latent clonality, including lymphoid one, promoting its clinical emergence (20).

Finally, 20% of patients developed a solid tumor, six after the second hematological malignancy and three in between. These cases had three neoplasms, and four of nine patients died. Although we did not find any association with previous therapies or hematological disease pattern, it may be speculated that an underlying genomic instability may be present in these cases (21).

We reckon that our study carries several limitations, particularly regarding the retrospective nature of the study, the relatively small number of patients, and the inclusion of diagnoses dating back to 30 years ago. However, only patients with overt clinical association and available detailed clinical data have been included, and all subjects have been followed at a single center and their diseases restaged/reclassified as per the state of the art. Larger multicenter studies including a control population would be important to confirm our observations.

In conclusion, our data suggest that in subjects with co-occurrence of myeloid and lymphoid neoplasms, high medical surveillance for infectious complications is needed, along with patient education, irrespective of treatment requirement, since they may negatively impact outcome.

## Data Availability Statement

The original contributions presented in the study are included in the article/**Supplementary Material**. Further inquiries can be directed to the corresponding author.

## Ethics Statement

Ethical review and approval was not required for the study on human participants in accordance with the local legislation and institutional requirements. Written informed consent for participation was not required for this study in accordance with the national legislation and the institutional requirements.

## Author Contributions

CB and BF followed patients, conceived the study, collected and analyzed data, and wrote the manuscript. DC, JG, KB, RP, EB, MC, LP, FR, GR, RC, WB, and LB followed patients and revised the article for important intellectual content. AI conceived and designed the study and supervised the analysis. All authors contributed to the article and approved the submitted version.

## Funding

The only funds used were those provided by the authors’ Institution.

## Conflict of Interest

The authors declare that the research was conducted in the absence of any commercial or financial relationships that could be construed as a potential conflict of interest.

## Publisher’s Note

All claims expressed in this article are solely those of the authors and do not necessarily represent those of their affiliated organizations, or those of the publisher, the editors and the reviewers. Any product that may be evaluated in this article, or claim that may be made by its manufacturer, is not guaranteed or endorsed by the publisher.
