# Supplementary material for: Co-Occurrence of Myeloid and Lymphoid Neoplasms: Clinical Characterization and Impact on Outcome. A Single-Center Cohort Study
Source: Front Oncol. 2021 Oct 18;11:701604. doi: 10.3389/fonc.2021.701604 (PMC8558405; doi:10.3389/fonc.2021.701604)
Supplement: Supplementary file 1 [file Table_1.docx]

**Supplementary Table: distributions of treatments and complications according to the sequence of myeloid/lymphoid neoplasm.** Years in brackets represent the time from the second hematologic neoplasm to the event. MF myelofibrosis; ET essential thrombocytemia; PV polycytemia rubra vera; MDS myelodysplastic syndrome; MPN-U myeloproliferative neoplasm unclassifiable.

| **Sequence** | **N.** | **First neoplasm** | **Therapy before 2^nd^ neoplasm** | **Second neoplasm** | **Therapy after 2^nd^ neoplasm** | **Thrombosis** | **Infections** | **Solid tumor** | **Death** |
| --- | --- | --- | --- | --- | --- | --- | --- | --- | --- |
| Myeloid first | 1 | MF | Ruxolitinib | PCD | Hydroxiurea | Yes (0.4 years after) | - | - | - |
|  | 2 | ET | Hydroxyurea | PCD | - | - | - | - | - |
|  | 3 | PV | Hydroxyurea | PCD | - | - | - | - | - |
|  | 4 | ET | Hydroxyurea | LPL | - | Yes (16 years before) | - | - | - |
|  | 5 | PV | - | PCD | Hydroxyurea | - | - | - | - |
|  | 6 | PV | Hydroxyurea | NHL | Pegylated interferon | - | - | - | - |
|  | 7 | MF | - | NHL | Radiotherapy | - | - | Yes (2 years before) | Yes |
|  | 8 | MF | Ruxolitinib | CLL | R-CHOP | - | - | - | - |
|  | 9 | ET | - | PCD | Hydroxyurea | - | - | - | - |
|  | 10 | MF | - | LPL | R-ibrutinib | - | - | - | - |
|  | 11 | ET | Hydroxyurea | PCD | - | - | - | - | - |
|  | 12 | ET | - | CLL | Hydroxyurea | Yes (17 years before) | - | - | - |
|  | 13 | PV | Hydroxyurea | LPL | - | - | - | Yes (14 years before) | - |
|  | 14 | PV | Hydroxyurea | LPL | - | - | Yes (2 years after) | - | - |
|  | 15 | ET | Hydroxyurea | CLL | - | Yes (7 years before) | Yes (0.2 year after) | Yes (1 year before) | - |
|  | 16 | MF | Hydroxyurea | NHL | R-bendamustine | Yes (1.5 years after) | Yes (1 year after) | - | - |
|  | 17 | PV | Hydroxyurea | NHL | R-COMP | - | Yes (2 years after) | - | Yes |
|  | 18 | LMC | Imatinib | PCD | Hydroxyurea, Lenalidomide dexamethasone | - | Yes (1 year after) | Yes (11 years after) | Yes |
|  | 19 | ET | - | NHL | Hydroxyurea, R-COMP | Yes (1.3 years before) | - | - | - |
|  | 20 | PV | - | CLL | Hydroxyurea,R-Bendamustine | - | - | - | - |
|  | 21 | MF | - | LPL | - | - | - | - | - |
|  | 22 | MPN-U | Hydroxyurea | NHL | Phototherapy | - | - | - | - |
|  | 23 | MF | Hydroxyurea, Pipobroman | CLL | Obinutuzumab- Chlorambucil | - | Yes (3 years after) | Yes (3 years after) | Yes |
| Concomitant | 24 | PV – LPL | - | - | Hydroxyurea | - | - | - | - |
|  | 25 | PV – CLL | - | - | Hydroxyurea, steroids | - | - | - | - |
|  | 26 | NOS – LPL | - | - | Hydroxyurea | - | - | - | - |
|  | 27 | PV – PCD | - | - | Hydroxyurea, Ruxolitinib | - | - | - | - |
|  | 28 | MDS/MPN – PCD | - | - | Hydroxyurea | - | - | - | - |
|  | 29 | MPN-U – LPL | - | - | Hydroxyurea | - | Yes (5 years after) | - | - |
|  | 30 | MDS – NHL | - | - | rEPO, steroids | - | - | - | - |
|  | 31 | MPN-U – LPL | - | - | Hydroxyurea | - | - | - | - |
|  | 32 | LMC – CLL | - | - | Imatinib | - | Yes (0.4 years after) | - | Yes |
|  | 33 | PV – PCD | - | - | Hydroxyurea | - | - | - | - |
|  | 34 | ET – CLL | - | - | Hydroxyurea | - | Yes (1 year after) | - | - |
|  | 35 | MF – CLL | - | - | Hydroxyurea | - | Yes (6 years after) | - | Yes |
|  | 36 | MDS – NHL | - | - | - | - | - | - | - |
|  | 37 | MF - NHL | - | - | Hydroxyurea | - | - | Yes (10 years after) | - |
| Lymphoid first | 38 | CLL | - | MF | Hydroxyurea, R-steroids | - | - | - | - |
|  | 39 | NHL | R-Fludarabine | MF | - | Yes (5 years before) | Yes (1 year before) | - | Yes |
|  | 40 | NHL | - | MF | Steroids | - | Yes (1 year after) | Yes (0.4years after) | - |
|  | 41 | CLL | - | NOS | Hydroxyurea, Chlorambucil | - | Yes (3 years after) | - | Yes |
|  | 42 | HL | ABVD | LMC | Hydroxyurea, Imatinib | - | - | Yes (3 years after) | - |
|  | 43 | NHL | Chlorambucil | MF | Hydroxyurea | - | - | Yes (5 years after) | Yes |
|  | 44 | NHL | Parotidectomy | PV | Hydroxyurea, Pipobroman | - | - | - | - |
